# Evaluation and treatment of latent tuberculosis infection among healthcare workers in Korea: A multicentre cohort analysis

**DOI:** 10.1371/journal.pone.0222810

**Published:** 2019-09-19

**Authors:** Seon-Sook Han, Su Jin Lee, Jae-Joon Yim, Jin Hwa Song, Eun Hye Lee, Young Ae Kang

**Affiliations:** 1 Department of Internal Medicine, Kangwon National University Hospital, School of Medicine, Kangwon National University, Chuncheon, Republic of Korea; 2 Department of Internal Medicine, Pusan National University Yangsan Hospital, Pusan National University School of Medicine, Yangsan, Republic of Korea; 3 Division of Pulmonary and Critical Care Medicine, Department of Internal Medicine, Seoul National University College of Medicine, Daehak-ro, Jongno-gu, Seoul, Republic of Korea; 4 Division of Pulmonology, Department of Internal Medicine, Severance Hospital, Yonsei University College of Medicine, Yonsei-ro, Seodaemun-gu, Seoul, Republic of Korea; Chinese Academy of Medical Sciences and Peking Union Medical College, CHINA

## Abstract

**Objective:**

Healthcare workers (HCWs) are one of the target groups for systematic testing and treatment of latent tuberculosis infection (LTBI) in a setting of low TB incidence. We performed this study to describe the testing of HCWs for LTBI and analyse the acceptance and completion of treatment of LTBI.

**Methods:**

This retrospective cohort study was conducted in four university-affiliated hospitals between January 1 and December 31, 2018. HCWs with positive interferon-gamma release assay (IGRA) during LTBI screening were analysed. We assessed the acceptance and completion of LTBI treatment.

**Results:**

Overall, 893 HCWs were IGRA positive. Among them, 609 HCWs visited the clinic for evaluation of LTBI. Of 609 HCWs who were evaluated, 302 (49.6%) were offered treatment for LTBI. The proportion of acceptance for treatment was 64.5% (195 of 302 HCWs). The treatment course was completed by 143 of 195 HCWs (73.3%). Three months of isoniazid and rifampin (3HR) was used in 137 HCWs (70.3%) and 4 months of rifampin (4R) in 58 (29.7%). 72 HCWs (36.9%) experienced at least one adverse drug events, but there was no different characteristics between completer and non-completer.

**Conclusion:**

The acceptance and completion of LTBI treatment were unsatisfactory. Subjective perspective regarding obstacles to treatment of LTBI needs to be explored to increase compliance to LTBI treatment.

## Introduction

The diagnosis and treatment of latent tuberculosis infection (LTBI) are important [1] for eliminating tuberculosis (TB). The prevention of active TB disease by treating LTBI is a critical component of the World Health Organization (WHO) End TB strategy [2].

Healthcare workers (HCWs) could be one of the target groups for the screening and treatment of LTBI because of the relatively high risk of developing active TB compared with the general population [3]. Based on the WHO 2018 guideline [4], HCWs are one of the target groups for systematic testing and treatment of LTBI in a setting of low TB incidence.

There are several obstacles to scaling up of LTBI treatment in HCWs. Traditionally, the acceptance of LTBI treatment among HCWs has been considered to be lower than that among non-HCWs [5, 6] with poor completion rates for a long duration of LTBI treatment [5–8]. Besides, it is important to exclude active TB before initiating treatment for LTBI. Symptomatic and radiographic screening has been used in the investigation of household contacts to exclude active TB [4]. A previous study revealed minor TB lesions on chest computed tomography (CT) in subjects with normal chest radiographs during contact investigation [9]. In the health care setting, exclusion of active TB is important because of possible transmission to patients.

This study aimed to assess the process of LTBI evaluation in HCWs and analyse the treatment acceptance and completion of 3–4 months of LTBI treatment. We also aimed to investigate treatment outcomes among HCWs in South Korea.

## Methods

### Study design and population

This retrospective cohort study was conducted in four university-affiliated hospitals in Korea. All HCWs from the four study hospitals who were positive for the interferon-gamma (IFN-γ) release assay (IGRA) during LTBI screening in 2017 as part of the national TB elimination program were enrolled [10, 11]. All participants who agreed to the testing of LTBI were screened between January 1 and December 31, 2017. HCWs who had positive IGRA results were enrolled in this study and followed up for the development of active TB until December 2018. The electronic medical records of these HCWs were retrospectively reviewed. We reviewed variables such as age, sex, body mass index (BMI), smoking, co-morbidities, and healthcare professions. The research protocol was approved by the institute review board of each hospital (approval numbers Institutional Review Board of Severance Hospital 4-2018-0960, Kangwon National University Hospital Institutional Review Board 2018-06-008-001, Pusan National University Yangsan Hospital Institutional Review Board 05-2019-044, Institutional Review Board of Seoul National University Hospital 1806-011-948). The requirement for informed consent was waived due to the retrospective nature of the study, in which all analyses used anonymous clinical data and presented minimal risk. Each institute review board approved the waiver of informed of consents.

### Evaluation for latent tuberculosis infection and tuberculosis

IGRA was performed using QuantiFERON-TB Gold In-Tube assay (Qiagen, Hiden, Germany) in accordance with the manufacturer’s instruction. A positive IGRA result was defined as an IFN-γ response to the TB antigen minus that of the Nil tube of ≥ 0.35 IU/ml. Symptom screening and chest radiographs were evaluated to exclude active TB at the same time. If a lesion suspicious of tuberculosis was seen on the chest radiograph, sputum examination and chest computed tomography (CT) were performed based on the discretion of the attending physician. Diagnosis of active TB was made based on all clinical, radiologic, and microbiological information. Active pulmonary TB was confirmed by the culture of M. tuberculosis from respiratory specimens, or by the presence of caseating granulomas in lung tissue or M. tuberculosis DNA. A culture-negative TB was defined for patients with high clinical likelihood of active TB, and a negative mycobacterial culture finding for two or more sputum examinations, but with good clinical and radiographic responses to anti-TB treatment during follow-up.

### Treatment of latent tuberculosis infection

HCWs who had positive IGRA results were advised to visit a pulmonary or infectious department out-patient clinic according to hospital policy. After medical evaluation and exclusion of active tuberculosis, the LTBI treatment recommendation and regimens were determined according to physician discretion. There was no case where HCW received treatment at a different hospital in this study. Treatment completion was defined as consumption of 80% of all prescribed medications without loss to follow-up [12, 13]. The treatment was not carried by directly observed treatment (DOT); instead, special TB nurses counseled and confirmed the patients with medication. During each medical appointment, attending physicians checked the patients' compliance to medications by asking how many pills patients had taken. All side effects (clinical and laboratory) were graded using the National Cancer Institute’s Common Terminology Criteria for Adverse Effects (CTCAE). All participants were followed up until the completion of LTBI treatment or loss to follow-up and data for the development of active TB were reviewed from medical records until December 2018.

### Statistical analysis

Continuous variables were reported as the median and range or interquartile range (IQR) and categorical variables as percentages and frequencies. Continuous variables were analysed using the Mann-Whitney U-test. Categorical variables were compared using the chi-square test or the Fisher’s exact test. Differences were considered statistically significant at P < 0.05 (two-tailed). All statistical analyses were performed using SPSS v18.0 (IBM, Armonk, NY, USA).

## Results

### Baseline characteristics of HCWs with positive IGRA results

**Fig 1:**
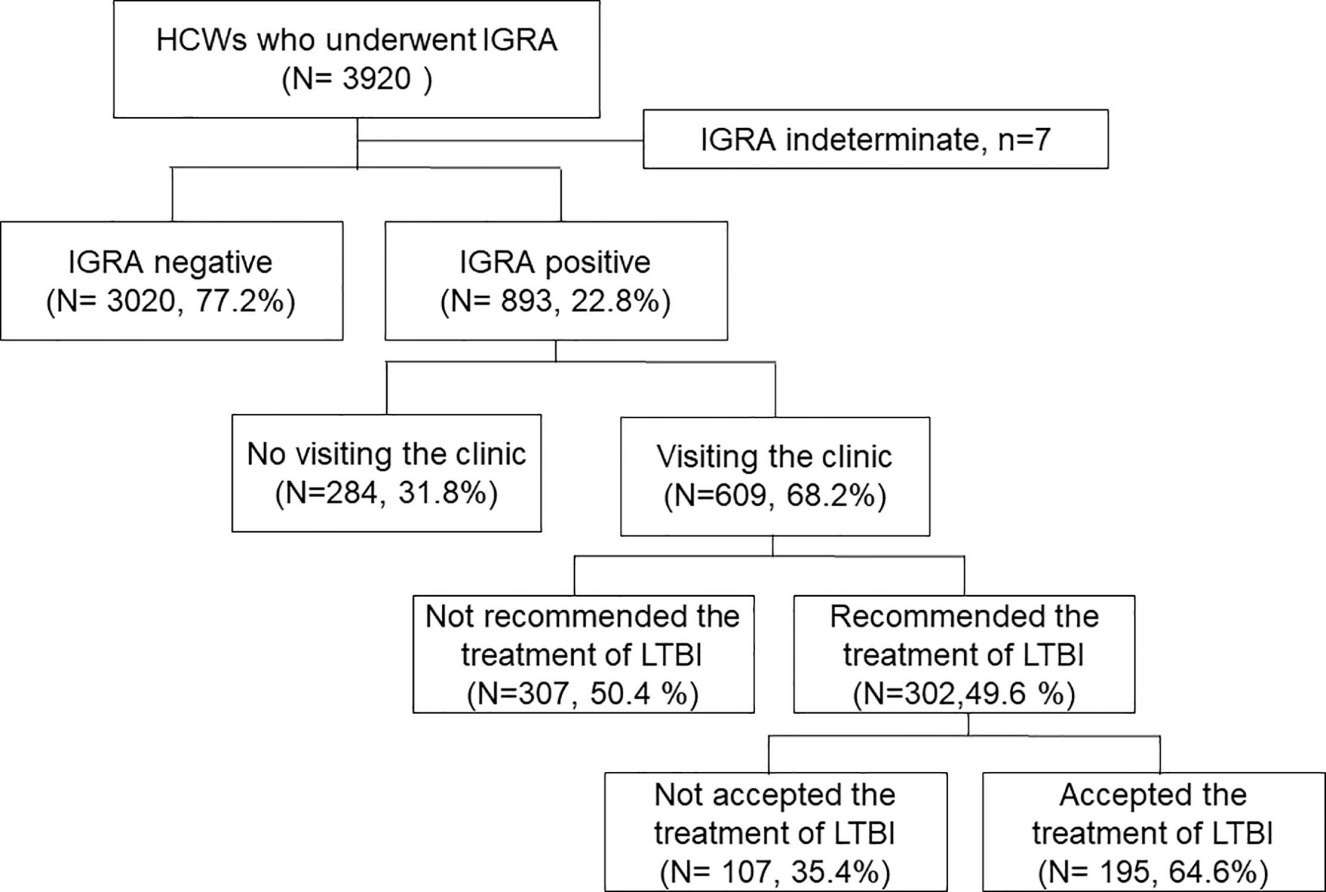
Flow chart of the study. HCWs, health care workers; IGRA, interferon-gamma release assay; LTBI, latent tuberculosis infection.

Overall, 3,920 HCWs underwent IGRA between January and December, 2017. Among them, 893 HCWs (22.8%) were positive for IGRA in the four study hospitals, including 654 in hospital A, 104 in hospital B, 112 in hospital C, and 23in hospital D. The median age of all IGRA-positive HCWs was 46 years (range 21–68), with 367 (41.1%) male subjects (Table 1). Of the 893 HCWs, 609 (68.2%) visited the clinic for evaluation of LTBI and active TB (Fig 1).

**Table 1:** Baseline characteristics IGRA positive HCWs.

	Total (N = 893)
Age (year), median, range	46 (21–68)
20–29, No (%)	78 (8.7)
30–39, No (%)	172 (19.3)
40–49, No (%)	372 (41.7)
50–59, No (%)	245 (27.5)
≥60, No (%)	25 (2.8)
Gender; Male, No (%)	367 (41.1)
Site	
Site A	654 (73.2)
Site B	104 (11.6)
Site C	112 (12.5)
Site D	23 (2.6)
BMI (kg/m^2^), median (IQR)	23.0 (21.0–25.0)
Smoking status^a^	
Never smoker	606 (74.1)
Ex-smoker	111 (13.6)
Current smoker	101 (12.3)
Profession	
Administrative	197 (22.1)
Technician^b^	92 (10.3)
Health aid^c^	266 (29.8)
Physician	99 (11.1)
Nurse	239 (26.8)
Work duration, month, median (IQR)	260 (117.7–315.8)
Comorbidities	
HTN	96 (10.8)
DM	34 (3.8)
History of TB treatment	58 (6.5)
Chest radiography	
No active lung lesion	873 (97.8)
Lesion suspicious of TB or others	20 (2.2)
Chest computed tomography performed	21 (2.4)
Active TB diagnosis	2 (0.2)
IFN- γ (TB Ag-Nil) concentration (IU/mL; median, IQR)	2.51 (0.92–6.13)

Data are presented as No (%) or median, range, and IQR.

^a^Data of Smoking were available for 818 HCWs.

^b^Technicians were defined as employees performing radiology, laboratory, or pathology-related duties.

^c^Health aides were defined as employees performing physiotherapy and patient transfer services.

HCWs, health care workers; BMI, body mass index; IQR, interquartile range; TB, tuberculosis; HTN, hypertension; DM, diabetes mellitus; IGRA, interferon-gamma release assay; IFN- γ, interferon gamma

### Exclusion of active TB in HCWs with positive IGRA

For the exclusion of active TB, chest radiographs were performed in all HCWs who were IGRA positive. Chest CT was carried out in 21 HCWs (2.4%) for the evaluation of abnormal lesions on the chest radiograph. No HCW had symptoms suggestive of TB, including cough for more than 2 weeks, fever, weight loss, and night sweats. During the medical evaluation, two HCWs were diagnosed with active pulmonary TB; one HCW was culture positive while the other was culture negative. These HCWs were treated with standard anti-TB medication. We calculated the sensitivity and specificity of chest X-ray for diagnosis of active TB. The sensitivity of the chest radiograph in for diagnosis of active TB was 50% (95% confidence interval, CI, 2.7% to 97.3) and the specificity was 97.9% (95% CI, 96.6% to 98.7%).

### Factors affecting the LTBI treatment recommendation by a physician

After exclusion of active TB, 302 HCWs (49.6%) were advised by physician to treat LTBI. Physician offered LTBI treatment less in those who were older, male, and with administrative profession. In multivariate regression analysis, physician offered LTBI treatment more in younger HCWs with profession of health aids and nurses (Table 2).

**Table 2:** Factors affecting the LTBI treatment recommendation by a physician.

	Visiting the clinic (N = 609)		
	Treatment offered (N = 302)	Bivariate analysis	Multivariate analysis
Age (year), median, range	42 (21–62)	0.94 (0.92–0.95)	0.975 (0.95–0.99)
Gender, female No (%)	210 (69.5)	1.69 (1.21–2.37)	1.00 (0.56–1.80)
BMI (kg/m^2^), median (IQR)	22 (20.8–24.7)	0.92 (0.84–0.97)	0.98 (0.92–1.05)
Never smoker^a^	228 (84.1)	2.05 (1.36–3.09)	1.12 (0.61–2.05)
HTN	18 (6.0)	0.52 (0.28–0.95)	0.92 (0.47–1.80)
DM	12 (4.0)	1.37 (0.56–3.30)	
Profession			
Administrative	38 (12.6)	reference	reference
Technician^b^	23 (7.6)	1.79 (0.93–3.47)	1.27 (0.61–2.64)
Health aid^c^	97 (32.1)	2.28 (1.42–3.64)	1.70 (1.00–2.87)
Physician	25 (8.3)	2.38 (1.19–4.53)	1.86 (0.90–3.85)
Nurse	119 (39.4)	5.23 (3.19–8.59)	3.43 (1.88–6.28)
Working duration, month, median (IQR)	237.4 (103.5–296.8)	0.99 (0.99–1.00)	
IFN-γ (TB Ag-Nil) concentration (IU/mL; median, IQR)	2.385 (0.878–5.865)	0.99 (0.97–1.02)	

Data are presented as No (%) or median, range, and IQR

^a^Data of Smoking were available for 572 HCWs.

^b^Technicians were defined as employees performing radiology, laboratory, or pathology-related duties.

^c^Health aides were defined as employees performing physiotherapy and patient transfer services.

LTBI, latent tuberculosis infection; BMI, body mass index; IQR, interquartile range; HTN, hypertension; DM, diabetes mellitus; IFN-γ, interferon gamma

Among 302 HCWs who were offered the treatment of LTBI, 195 HCWs (64.6%) accepted the physician’s recommendation. When we compared the characteristics of HCWs between acceptors and decliner of LTBI treatment, acceptors were younger than decliners.

### Comparison of the characteristics between HCWs who completed treatment and those who did not

Among 195 HCWs who commenced LTBI treatment, 143 (73.3%) completed the course of treatment. We did not find any specific risk factors for non-completion of LTBI treatment (Table 3). There was no difference in age, gender, profession, and duration of employment between those who completed treatment and those who did not. 137 HCWs (70.3%) were treated with 3 months of isoniazid and rifampin (3HR) and 58 (29.7%) with 4 months of rifampin (4R). There was no difference between the two regimens in completion of treatment. More than one-third of HCWs (36.9%) developed adverse drug events during treatment. There was no difference in the incidence of adverse events between those who completed (35%) and those who did not complete treatment (42.3%). However, HCWs who experienced the flu-like symptoms more likely stopped the medication for LTBI (Table 3). Among 52 HCWs who did not complete treatment, 22 (42.3%) stopped treatment due to adverse drug events.

**Table 3:** Comparison of the characteristics between HCWs who completed LTBI treatment vs. those who did not.

Characteristic	Total (N = 195)	Completed (N = 143)	Did not complete (N = 52)	*P*
Age (year), median, range	40 (21–62)	41 (21–59)	39 (23–62)	0.803
Gender,female, No (%)	138 (70.8)	101 (70.6)	37 (71.2)	0.943
BMI (kg/m^2^), median (IQR)	22 (21–24)	22 (21–24)	22 (20–24)	0.421
Never smoker^a^	150 (88.8)	109 (87.2)	41 (93.2)	
HTN	11 (5.6)	9 (6.3)	2 (3.8)	0.731
DM	7 (3.6)	5 (3.5)	2 (3.8)	1.000
Profession				0.544
Administrative	17 (8.7)	12 (8.4)	5 (9.6)	
Technician^b^	26 (8.2)	9 (6.3)	7 (13.5)	
Health aid^c^	65 (33.3)	48 (33.6)	17 (32.7)	
Physician	18 (9.2)	13 (9.1)	5 (9.6)	
Nurse	79 (40.5)	61 (42.7)	18 (34.6)	
Work duration, month, median (IQR)	184.2 (65.5–290.7)	181.9 (69.8–287.1)	186.2 (29.0–296.8)	0.952
Any adverse drug events	72 (36.9)	50 (35.0)	22 (42.3)	0.347
Abnormal liver function^d^	18 (9.1)	13 (9.1)	5 (9.6)	1.000
Fatigue	28 (14.4)	19 (13.3)	9 (17.3)	0.479
Gastrointestinal symptoms	19 (9.7)	12 (8.4)	7 (13.5)	0.291
Rash	12 (6.2)	7 (4.9)	5 (9.6)	0.309
Pruritus	9 (4.6)	7 (4.9)	2 (3.8)	1.000
Flu-like symptoms	8 (4.1)	2 (1.4)	6 (11.5)	0.005
Regimen				
3HR	137 (70.3)	100 (69.9)	37 (71.1)	0.869
4R	58 (29.7)	43 (30.1)	15 (28.8)	

Data are presented as No (%) median, range, and IQR.

^a^Data of Smoking were available for 169 HCWs.

^b^Technicians were defined as technical employees performing radiology, laboratory, or pathology-related duties.

^c^Health aids were defined as employees performing physiotherapy and patient transfer services.

^d^Abnormal liver function means an elevation of alanine/aspartate aminotransferase by more than three times the upper limit of normal range if baseline was normal based on CTCAE version 5.0.

LTBI, latent tuberculosis infection; HCWs, health care workers; BMI, body mass index; IQR, interquartile range; HTN, hypertension; DM, diabetes mellitus; 3HR, 3 months of isoniazid and rifampin; 4R 4 months of rifampin.

**Fig 2:**
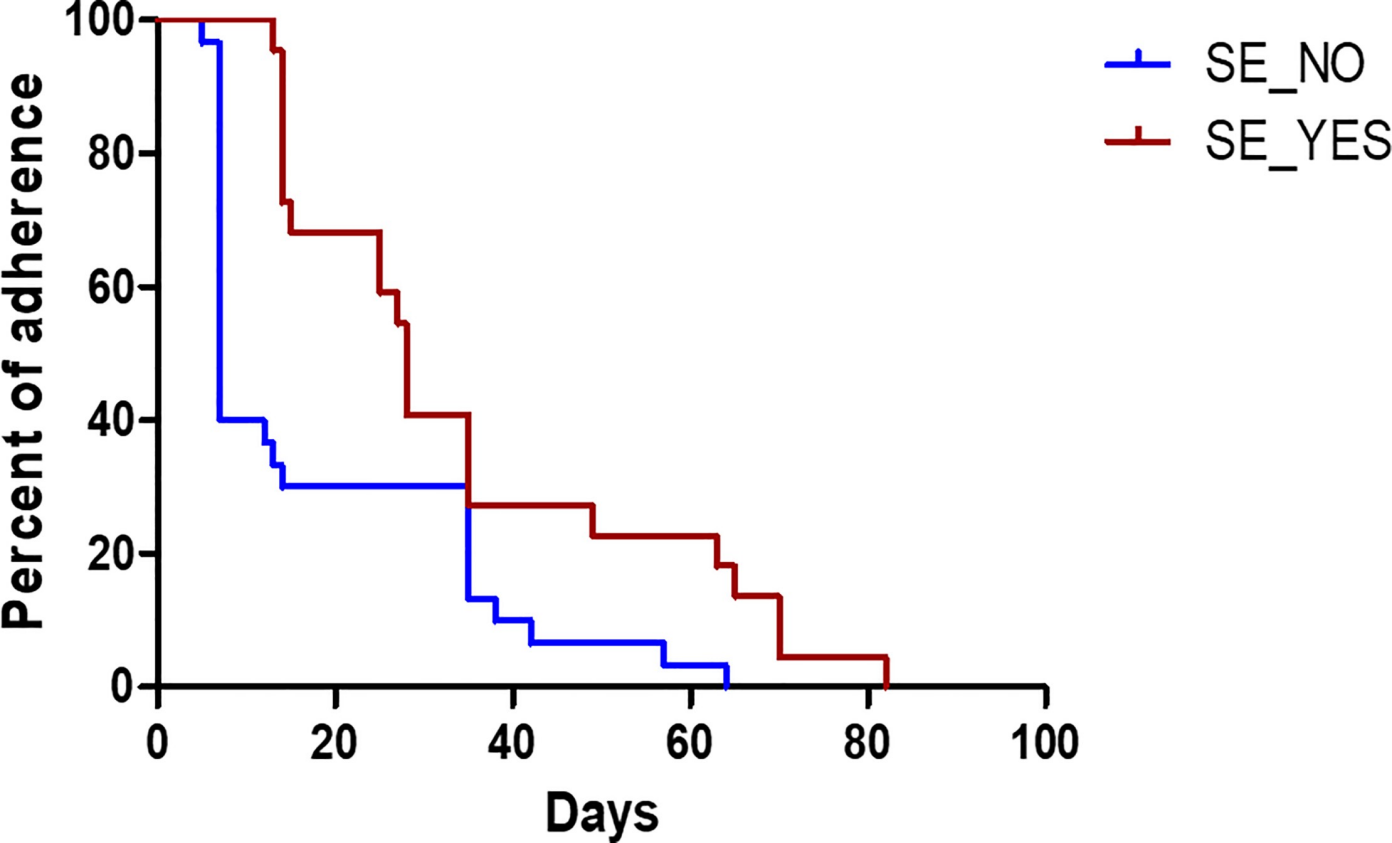
Drop out of LTBI treatment among 52 healthcare workers who did not complete treatment. Among the 52 HCWs who did not complete treatment, most dropped out in the early phase of treatment. In the first 2 weeks, 51.9% HCWs dropped out; 80% of those who did not complete treatment did not present for follow up after 5 weeks. SE, side effect.

Among those who failed to complete the treatment course, the majority of HCWs dropped out in the early phase of treatment (Fig 2). More than a half of those who failed to complete the treatment course (51.9%) dropped out within 2 weeks and 80% of those who dropped out did not present for follow-up in the clinic at 5 weeks after commencement of treatment.

### Comparison of the characteristics of HCWs according to the occurrence of ADRs

For the treatment of LTBI, 72 HCWs (36.9%) experienced at least one ADR. The characteristics were not different between HCWs with ADR and those without (Table 4). There was no difference between the two regimens (3HR vs 4R) in the development of ADRs.

**Table 4:** Comparison of the characteristics between HCWs who experienced ADRs or not.

Characteristic	Total (N = 195)	ADRs negative (N = 123)	ADRs positive (N = 72)	*P*
Age (year), median, range	40 (21–62)	38.9 (29.3–47)	42 (34.8–47.5)	0.100
Gender; female, No (%)	138 (70.8)	88 (71.5)	50 (69.4)	0.756
BMI (kg/m^2^), median (IQR)	22.0 (21.0–24.0)	22.4 (20.8–24.9)	22.0 (20.0–24.0)	0.138
Never smoker^a^	150 (88.8)	95 (89.6)	55 (87.3)	0.644
HTN	11 (5.6)	4 (3.3)	7 (9.7)	0.103
DM	7 (3.6)	3 (2.4)	4 (5.6)	0.427
Profession				0.061
Administrative	17 (8.7)	7 (5.7)	10 (13.9)	
Technician^b^	26 (8.2)	13 (10.6)	3 (4.2)	
Health aid^c^	65 (33.3)	36 (29.3)	29 (40.3)	
Physician	18 (9.2)	13 (10.6)	5 (6.9)	
Nurse	79 (40.5)	54 (43.9)	25 (34.7)	
Work duration, month, median (IQR)	184.2 (65.5–290.65)	153.7 (40.7–282.7)	213.1 (93.6–294.7)	0.325
Regimen				0.607
3HR	137 (70.3)	88 (71.5)	49 (68.1)	
4R	58 (29.7)	35 (28.5)	23 (31.9)	
Completion of treatment	143 (73.3)	93 (75.6)	50 (69.4)	0.347

^a^Data of Smoking were available for 169 HCWs.

^b^Technicians were defined as technical employees performing radiology, laboratory, or pathology-related duties.

^c^Health aids were defined as employees performing physiotherapy and patient transfer services.

ADRs, adverse drug reactions; HCWs, health care workers; BMI, body mass index; IQR, interquartile range; HTN, hypertension; DM, diabetes mellitus; 3HR, 3 months of isoniazid and rifampin; 4R 4 months of rifampin.

## Discussion

We analysed the LTBI evaluation process and treatment outcomes regarding acceptance and completion of treatment among HCWs.

HCWs are at high risk for developing active TB and LTBI compared to the general population [3]. TB among HCWs is important because they are at risk of developing infection from the work place. Besides, HCWs may transmit infection to patients in health care settings. Therefore, regular screening for TB and LTBI among HCWs has been previously conducted in low incidence settings [14, 15], although there were several controversial issues including cost-effectiveness [16, 17]. Furthermore, the diagnosis and treatment of LTBI among HCWs in settings of intermediate burden such as South Korea may help with the control of TB in health care settings.

A significant number of HCWs did not visit the clinic for evaluation posing a major hindrance to systematic screening for LTBI and TB. In this study, only 68.2% of HCWs underwent evaluation. Exclusion of active TB by symptom screening and chest radiography may not be adequate in some cases. In the present study, all HCWs underwent chest radiography. Active TB was diagnosed on chest CT and clinical situation in one HCW who was asymptomatic and had no suspicious lesions on the chest radiograph. He had minor findings suggestive of active TB on the chest CT and was culture negative for mycobacteria. He was diagnosed and treated based on the clinical judgement of the attending physician. Pooled sensitivity and specificity of symptom screening and abnormal chest radiograph to rule out active TB in subjects with human immunodeficiency virus (HIV) infection on anti-retroviral therapy (ART) was 89.3% (95% CI, 82.6–93.6) and 27.2% (95% CI, 17.3–40.0) [4] in a previous report. Thus, in some cases, the sensitivity and specificity of chest radiography and symptom screening are not enough for the exclusion of active TB in the intermediate and high burden setting. Physicians need to exercise caution and exclude active TB before the commencement of treatment for LTBI in these settings.

The low acceptance rate (64.6%) for LTBI treatment among HCWs was another impediment in the present study, consistent with previous reports [18]. One-third of the Korean population were infected with *M*. *tuberculosis* bacilli, and the infection rate increased with age [19]. IGRA screening among employees aged more than 60 years working in congregated facilities showed more than 40% positive for IGRA in South Korea [10]. Besides, the incidence of adverse events increased with age. Thus, many HCWs in the older age group were reluctant to undergo treatment for LTBI. In the present study, we found a variation in the acceptance for the treatment of LTBI between study sites ranging from 52% to 98%. The wide range of acceptance in our study implies the possibility of different target populations among HCWs and variable levels of recommendation by attending physicians at study sites.

The completion rate of LTBI in this study was 73.3% which is comparable to previous reports [17, 20]. The absence of difference in characteristics between subjects who completed treatment and those who did not was surprising. Among those who did not complete treatment, 22 HCWs (42.3%) stopped treatment due to adverse events, while the rest (57.7%) dropped out without specific reasons. The relatively early dropout among those who did not complete treatment (Fig 2) may imply that HCWs accepted the LTBI treatment initially, but were unsure about continuing the full course of treatment.

Shorter regimens for LTBI have been developed to improve treatment compliance compared to the 9-month isoniazid regimen (9H). Recently, 12 doses of rifapentine and isoniazid have been recommended [21]; besides, 4R has shown comparable efficacy to 9H [12]. In the present study, HCWs were treated with 3HR or 4R according to physician discretion. There was no difference in completion and the occurrence of adverse events between two regimens.

There are several limitations to the present study. Although this was a multicentre study, only 4 centres were involved. All HCWs in this study were enrolled in the national TB elimination program, which was conducted in accordance with the Tuberculosis Prevention Act. Therefore, we could not generalize these results in other settings for evaluation of LTBI in HCWs. There were several missing data in basic characteristics, including smoking, co-morbidity and history of TB exposure. In addition, we could not assess the real barriers for acceptance and completion of treatment from the perspective of the HCW’s. Hence, qualitative research is required to further explore the obstacles to treatment from the perspective of subjects.

## Conclusions

In conclusion, the acceptance and completion of LTBI treatment were unsatisfactory. However, the factors affecting the completion of treatment was not clear. For scaling up of screening and treatment of LTBI, qualitative research to explore the subjective personal obstacles may be helpful.

## Supporting information

S1 Dataset DATA_HCW_201908_supporting information.xlsx. This is the dataset. (XLSX)

S2 Dataset DATA _HCW_Dictionary.xlsx. This is the dataset dictionary.
(XLSX)
